# Effect of physical exercise on metabolism in patients with atrial fibrillation

**DOI:** 10.3389/fcvm.2024.1502620

**Published:** 2024-12-19

**Authors:** Yutong Wang, Sixian Weng, Chenxi Xia, Tao Xu, Xinyang Song, Fang Wang

**Affiliations:** ^1^ Cardiology Department, Beijing Hospital, National Center of Gerontology, Institute of Geriatric Medicine, Chinese Academy of Medical Sciences & Peking Union Medical College, Beijing, China; ^2^ Department of Cardiology, Beijing Anzhen Hospital, Affiliated to Capital Medical University, Beijing, China; ^3^ Cardiology Department, Beijing Hospital, Peking University Fifth School of Clinical Medicine, Beijing, China; ^4^ Cardiology Department, Beijing Hospital, National Center of Gerontology, Beijing, China

**Keywords:** physical exercise, atrial fibrillation, metabolism, physical activity, metabolic dysfunction

## Abstract

Atrial fibrillation (AF), the most prevalent cardiac arrhythmia, is closely linked to metabolic dysfunctions, including obesity, diabetes, and dyslipidemia. These lead to pathological changes in myocardial metabolism and mitochondrial energy metabolism, thereby aggravating AF's incidence and severity. This review introduces the role of metabolic dysfunctions in exacerbating AF, assesses the therapeutic potential of physical exercise and investigates it as a non-pharmacological intervention to alleviate these metabolic disturbances. Evidence suggests that regular physical activity not only enhances metabolic profiles but also reduces the frequency of AF episodes and improves overall cardiovascular health. At the same time, the review emphasizes the need for individualized exercise regimens, individualized to the metabolic and cardiac conditions of each patient to optimize benefits and minimize risks. Additionally, it calls for more basic studies and large-scale clinical trials to establish and refine evidence-based exercise guidelines specific to AF management.

## 1 Introduction

Atrial fibrillation (AF) is a prevalent arrhythmia affecting 1% of individuals over the age of 60. The estimated global prevalence was 50 million in 2020. The main risk factors for AF include obesity, physical inactivity, diabetes and high blood pressure. As these factors are exacerbated with age, their incidence gradually increases with age. The 2023 ACC/AHA Guideline for the Management of AF shows that the increasing burden of AF is multifactorial, including the aging of the population, and a rising tide of obesity. The latest ESC guidelines for the management of atrial fibrillation in 2024 show that the prevalence of AF is expected to double in the coming decades due to an aging population, increasing comorbidities, and other reasons (1). To achieve optimal treatment of patients with AF, it is now widely recognized that early and dynamic management of these comorbidities and risk factors is necessary.

Metabolism refers to the organized series of chemical reactions essential for sustaining life, divided into material metabolism and energy metabolism. Material metabolism, encompassing anabolism and catabolism, is primarily the process by which the three major nutrients, sugar, lipids, and proteins, are digested, absorbed, operated on, and broken down in the body. Material metabolism is often accompanied by energy transformations, and catabolism often releases energy. The heart needs large amounts of energy to support its pumping, and the main form of energy, ATP, is obtained mainly from glucose, fatty acids and amino acids in the blood. Therefore, a sophisticated and efficient energy metabolism that works in tandem with material metabolism to maintain myocardial function. Metabolic disorders affecting the production of energy required to maintain systolic function and cardiac output may underlie disease entities.

Atrial fibrillation is marked by the rapid excitation and contraction of atrial myocytes, often associated with ischemia and hypoxia in tissues and organs. In this context, alterations in metabolites can serve as a compensatory mechanism (2). Research indicates that myocardial metabolism plays a crucial role in the pathophysiology of atrial fibrillation (3), and metabolic disorders directly influence the development of atrial fibrillation substrates (4). Thus, the onset and progression of atrial fibrillation are intimately linked to human metabolism and mitochondrial function.

Metabolic disorders including obesity, hyperlipidemia, metabolic syndrome, and diabetes are recognized risk factors for cardiometabolic diseases such as atrial fibrillation and heart failure. Additionally, the age of patients and their medications often contribute to a complex *in vivo* metabolic state, further disrupting metabolic equilibrium (5). A national study focusing on Asian populations revealed that individuals with metabolic health issues face a significantly increased risk of atrial fibrillation compared to those who are metabolically healthy (6).

As health awareness increases, particularly in developing countries, greater emphasis is being placed on physical activity. This not only aids in heart function and mood regulation but also appears to slow the progression of atrial fibrillation in patients. Although the specific benefits, such as improvements in blood metabolites, cardiac remodeling, and electrophysiological function, are not fully understood, the evidence underscores the need for further, individualized research to target physical activity recommendations effectively.

This review explores the altered metabolic status in patients with atrial fibrillation, with a focus on energy and substance metabolism. It aims to assess how exercise can improve symptoms and enhance the quality of life, analyzing the underlying mechanisms through which physical activity impacts patients with atrial fibrillation.

## 2 Metabolic characteristics of patients with atrial fibrillation

### 2.1 Lipid metabolism and atrial fibrillation

Under normal conditions, the heart generates ATP primarily from glucose, fatty acids, and amino acids, which it must derive from the bloodstream due to its limited storage capacity. Notably, over 50% of the ATP produced in the mitochondria is derived from fatty acid oxidation (7). Within cardiomyocytes, ingested fatty acids are converted into fatty acyl-coenzyme A (CoA), which is then subjected to *β*-oxidation to produce acetyl-CoA. This acetyl-CoA enters the tricarboxylic acid (TCA) cycle, essential for substance metabolism. Concurrently, this process generates FADH2 and NADH, which are crucial for the electron transport chain in the mitochondrial membrane, facilitating ATP production. Changes in the metabolites associated with the TCA cycle indicate cardiac metabolic dysregulation, often due to altered hemodynamics, hypoxia, and insufficient energy substrates. Obesity is a known risk predictor for atrial fibrillation, as the accumulation of fat enhances neurohormonal activation, oxidative stress, and cardiac remodeling (8). Obesity, a pro-inflammatory state, heightens the risk of insulin resistance, hypertension, and hyperlipidemia due to adipose tissue enlargement. The location of this enlargement varies in risk. Ectopic adipose tissue, such as myocardial adipose near coronary arteries, epicardial adipose tissue (EAT), and pericardial adipose tissue (PAT), significantly increases cardiovascular risk. This is particularly critical in patients with type 2 diabetes, where it contributes to structural and energetic abnormalities in the heart (9). EAT releases pro-inflammatory and pro-fibrotic cytokines such as IL-1β and IL-6 via paracrine or vascular secretion, with deleterious effects such as accelerated vascular sclerosis, a condition that can be mitigated through medication and physical activity (10). In addition, EAT and PAT play a role in the pathogenesis of cardiovascular diseases such as AF through complex mechanisms that include gene expression profiles, neuromodulation, and glucose and lipid metabolism (11). Specifically, EAT and PAT have become a risk factor and independent predictors of AF occurrence and recurrence after ablation (12, 13). The infiltration of EAT into the atrial myocardium plays a crucial role in forming the substrate for atrial fibrillation. The pathogenic role of EAT in AF may begin during embryogenesis and development, affecting the differentiation of cardiac fibroblasts and smooth muscle cells. Fatty acid infiltration and fibrosis then lead to cardiac conduction abnormalities and affect the local electrophysiological properties of the atria (11, 14). An Australian clinical study demonstrated that obesity leads to increased EAT infiltration, which in turn promotes atrial fibrosis and disrupts normal cardiac electrophysiological conduction (15). Patients with fatty liver exhibit thicker epicardial adipose tissue and more severe fibrosis compared to healthy individuals (16). Studies have established a connection between nonalcoholic fatty liver disease (NAFLD) and atrial fibrillation, noting significant changes in heart structure and function, including diastolic dysfunction. Furthermore, cardiometabolic risk factors often interact, exacerbating the adverse effects of fat accumulation, particularly in hypertensive patients (17).

In obese patients, those with metabolic disorders—such as hypertension, hypercholesterolemia, or diabetes—are more prone to the progression of atrial fibrillation (18). These metabolic disorders contribute to impaired vascular function, cellular inflammation, fibrosis, and remodeling of the atrial and arterial walls. This indicates that metabolic conditions are interconnected, with one abnormality potentially exacerbating others, leading to more severe health consequences.

Lipidomic and metabolomic analyses of obese mouse models on prolonged high-fat diets reveal that obesity disrupts atrial energy metabolism, primarily through the accumulation of long-chain lipids and the activation of β-oxidation. The excessive influx of fatty acids into the atria modifies the oxidative profile of the atrial myocardium, resulting in fatty, inflamed tissue and altered electrical properties, thereby increasing susceptibility to atrial fibrillation (19).

### 2.2 Glucose metabolism and atrial fibrillation

Poor glycemic control exacerbates oxidative stress, chronic inflammation, and leads to cardiac hypertrophy, as well as increased vascular and interstitial collagen deposition. It also contributes to abnormal intracellular calcium levels, endothelial and mitochondrial dysfunction, and increased apoptosis. Consequently, individuals with diabetes, particularly those with poor glycemic control and albuminuria, face a significantly higher risk of atrial fibrillation compared to the general population (20). Fibrosis development in the myocardial interstitium impairs cardiac electrophysiological conduction, a condition notably exacerbated in diabetic patients.

Hyperglycemia, a state of metabolic dysregulation, can induce stress in the endoplasmic reticulum, an organelle crucial for protein synthesis, folding, and transport. Under stress, it releases calcium into the cell, which then enters the mitochondria through calcium channels, inducing oxidative stress and impacting energy metabolism. Inhibiting this calcium transfer between the endoplasmic reticulum and mitochondria can mitigate calcium overload and oxidative stress, reduce the generation of reactive oxygen species (ROS) in mitochondria, and decrease apoptosis (21).

Diabetes mellitus and cardiovascular diseases are linked by numerous pathogenic mechanisms. At the vascular level, diabetes-related complications often present as pan-vascular lesions, characterized by endothelial dysfunction, which frequently leads to cardiovascular events. Diabetes also disrupts cardiac metabolism through glucotoxicity and lipotoxicity, impairs mitochondrial function, and triggers inflammation. These factors contribute to endoplasmic reticulum stress and apoptosis, ultimately leading to ventricular remodeling (22). Individuals with elevated blood glucose levels are at an increased risk of developing atrial fibrillation, particularly its persistent and permanent forms. Diabetes is not merely an isolated condition but part of a complex systemic disorder. This complexity is heightened as hyperglycemic individuals frequently exhibit comorbidities such as hypertension, peripheral vascular disease, obesity, and thromboembolism, all of which significantly contribute to the onset of atrial fibrillation (23).

Antidiabetic drugs are not only beneficial in controlling blood sugar but also have a protective effect on the heart. Dipeptidyl peptidase-4 (DPP-4) inhibitors ameliorate glucose metabolism by enhancing glucagon-like peptide-1 (GLP-1) receptor signaling, inducing insulin secretion and inhibiting glucagon secretion (24). DPP-4 inhibitors as well as GLP-1 receptor agonists can reduce monocyte/macrophage accumulation by inhibiting the inflammatory response of monocytes/macrophages and also beneficial to the cardiovascular system (25–28). Alogliptin, a DPP-4 inhibitor commonly used in diabetes management, not only regulates blood glucose levels but also exhibits antioxidant, anti-inflammatory, and antifibrotic properties, offering cardioprotective benefits (29, 30). Sodium-dependent glucose transporters 2 inhibitors(SGLT2i), which inhibit glucose reabsorption by the kidneys, allowing excess glucose to be excreted in the urine and reducing blood glucose. As a new antidiabetic drug, it also shows positive cardioprotective properties and can reduce hospitalization for cardiovascular outcomes in patients (31). Patients with type 2 diabetes at high risk for cardiovascular events who were treated with empagliflozin had a reduced incidence of the major composite cardiovascular outcome and all-cause mortality (32). Large-scale clinical trial has shown a decrease in the incidence and recurrence of atrial fibrillation in people treated with dapagliflozin (33). The largest network meta-analysis published to date has shown that the use of gliflozins has a significant effect on the reduction of the incidence of atrial fibrillation in the overall population (34).

### 2.3 Mitochondrial function and atrial fibrillation

Mitochondria are central to several cellular functions, including energy production (ATP), ROS generation and scavenging, calcium homeostasis, and regulation of nuclear gene expression, as well as cell death and survival mechanisms. As the main site for ROS production, maintaining mitochondrial integrity is vital for cardiac health. Cardiac tissues generate ROS through mechanisms involving NADPH oxidase, xanthine oxidase, and uncoupled nitric oxide synthase. Damage to mitochondrial DNA or mitochondrial dysfunction can disrupt cardiac rhythms by impairing the energy supply essential for ion channels and transporters (35, 36). Elevated levels of reactive oxygen species (ROS) can damage proteins, lipids, and DNA, particularly mitochondrial DNA. Additionally, ROS promotes inflammation by enhancing cytokine production and activating inflammatory cells, which exacerbates tissue damage. Increased mitochondrial ROS production is also associated with the formation of arrhythmic substrates, contributing to both structural changes and electrocardiographic remodeling in the heart. The dynamic balance of mitochondrial fusion and fission, which regulates mitochondrial size and quantity, is crucial for maintaining mitochondrial function. Disruption in this balance can impair the energy supply required for cardiac activity, leading to abnormal cardiac function.

Evidence suggests that mitochondrial dysfunction in the atria plays a critical role in the development of atrial fibrillation. Atrial remodeling, which includes structural and functional changes such as alterations in electrical properties and contractility, along with changes in the extracellular matrix composition, promotes tissue heterogeneity. This heterogeneity can lead to slowed conduction and electrolytic uncoupling, further advancing atrial fibrillation. Key mechanisms driving atrial remodeling include impaired mitochondrial oxidative respiration and endoplasmic reticulum stress. Additionally, inflammation and oxidative stress are pivotal in the pathogenesis of atrial fibrillation, highlighting the potential effectiveness of antioxidant therapies in preventing atrial remodeling (37). Research has demonstrated that alogliptin reduces mitochondrial swelling in the atria of diabetic animal models, stabilizes mitochondrial membrane potential, and curbs ROS production. Additionally, it prevents mitochondrial respiratory dysfunction and electrical remodeling of the heart (38). Furthermore, alogliptin reduces insulin resistance and plasma stress, and it restores impaired mitochondrial function (39).

### 2.4 Alcohol, caffeine, and cardiac electrophysiology

Caffeine and alcohol are known to trigger acute episodes of atrial fibrillation. Smoking increases the risk of AF by more than twofold, while the incidence tends to decrease in those who quit smoking (40). These correlation may be influenced by blood metabolites and cardiac electrophysiological conduction (41).

### 2.5 Renal metabolism and atrial fibrillation

The kidneys are essential organs for metabolism, playing a crucial role in maintaining body fluid and electrolyte balance, as well as performing endocrine functions. The incidence of atrial fibrillation is notably high in patients with chronic kidney disease. Chronic kidney disease and elevated urinary albumin-to-creatinine ratios are associated with the development of atrial fibrillation (42). Additionally, an association has been observed between chronic kidney disease and the incidence of atrial fibrillation in participants with a mean age over 70 years (43). Therefore, it is especially important to focus on the relationship between kidney health and cardiovascular health in older adults.

### 2.6 Gut flora and atrial fibrillation

Intestinal flora, composed of the normal microorganisms in the human gut, play a critical role in metabolizing sugars and proteins, as well as absorbing trace elements. The metabolism of intestinal flora is linked to the development of cardiovascular disease. Regular physical activity not only affects the distribution and metabolites of intestinal flora but also enhances intestinal barrier function. Promoting a healthy gut flora phenotype reduces the risk of cardiovascular diseases. Although the specific mechanisms are not fully understood, they may involve processes such as cellular material exchange, energy production, endotoxemia, bacterial translocation, and metabolite accumulation (44, 45). Physical activity helps regulate the transition of gut flora to a healthy phenotype, producing inflammatory factors that may modulate intestinal permeability and influence cardiovascular physiology (46).

## 3 Mechanisms of the effect of physical activity on atrial fibrillation

Atrial fibrillation is characterized by cardiac electrophysiological dysregulation, with patients experiencing abnormalities in the expression and activity of ion channels in cardiomyocytes. These changes may be related to fibroblast-mediated stress and mechanical stimulation. Elevated blood levels of inflammatory factors, such as IL-6, may also serve as potential mechanical stimuli, influencing ion channel activity in atrial myocytes (47). During exercise, the release of catecholamines, specifically epinephrine and norepinephrine, increases heart rate and cardiac contractility. This is accompanied by skeletal muscle vasodilation, muscle congestion, and enlargement. In individuals who engage in long-term exercise training, the myocardium may become hypertrophied due to increased cardiac load, driven by sympathetic nerve activation and the release of growth factors. However, this type of hypertrophy differs from pathological hypertrophy, as it lacks pathological features like fibrosis (48).

Animal experiments have shown that regular exercise training inhibits the expression of potassium channels and prolongs the atrial operational period, which protects the myocardium in atrial fibrillation (AF). Conversely, enhanced parasympathetic activity from strenuous exercise activates acetylcholine-sensitive potassium channels, shortening the refractory period and increasing susceptibility to AF (49).

Study has demonstrated that exercise training improves exercise capacity, cardiac function, and quality of life in AF patients (50). Patients undergoing catheter ablation for AF often show improved exercise capacity due to better cardiac rhythm control. However, a lack of improvement in exercise capacity within 12 months post-ablation is an independent risk factor for AF recurrence (51). The UK Biobank study, involving nearly half a million people, found that physically active participants had a lower risk of AF and reduced risk of ventricular arrhythmias (52). A European cohort study on AF outcomes revealed a nearly 35% increase in the odds of AF progression in physically inactive patients, with a higher incidence of AF progression at 1-year follow-up compared to active patients (17.7% vs. 6.8%) (53). Additionally, exercise-based rehabilitation reduced rates of all-cause mortality, rehospitalization, and stroke, regardless of gender, age, or AF subtype (54). Baseline survey has shown that physical activity in AF patients is associated with lower mortality, independent of gender and age (55). The risk of AF is lower in the exercise maintenance group compared to the continuously inactive group. An exercise level of 1,500 MET-min/week (1 MET equals 1 kilocalorie of energy burned per kilogram of body weight per hour) is the threshold above which exercise reduces the risk of AF progression. Current guidelines recommend 150 to 300 min of moderate-intensity aerobic exercise, 150 min of higher-intensity aerobic exercise, or 75 min of vigorous exercise per week, equivalent to 450 METs of physical activity (56). A meta-analysis including 1,464,539 people showed that achieving guideline-recommended exercise levels effectively reduces the risk of AF (57). Regular-intensity training improves risk factors for AF such as sleep apnea syndrome, obesity, and hypertension, and also reduces the burden of AF (58). Another study of 10,000 people confirmed that completing cardiac rehabilitation alone had no significant effect on reducing AF risk, highlighting the importance of improving cardiorespiratory fitness (59). Moderate physical activity and enhanced cardiorespiratory fitness have been associated with a lower long-term risk of cardiovascular disease and all-cause mortality in AF patients. This finding, from cohort studies, supports the role of routine physical activity and improved cardiorespiratory fitness in reducing the elevated risk of mortality and morbidity in AF patients (60).

However, it is important to note that greater exercise intensity does not always equate to greater benefits. Current consensus suggests that, within limits, more intense exercise improves cardiorespiratory fitness. Yet, strenuous high-intensity exercise can be harmful (61), as it increases the risk of sudden death, acute myocardial infarction, stroke, and ventricular arrhythmia (62). High-intensity physical activity can lead to inflammation, remodeling, and autonomic activation (63), and the inflammatory environment resulting from prolonged intense exercise may trigger harmful arrhythmic events (64). The aforementioned meta-analysis indicated that the risk reduction for atrial fibrillation ceases when exercise exceeds 1,900 MET minutes per week (57). Strenuous endurance exercise can induce adverse atrial remodeling and increase arrhythmic susceptibility. In animal experiments, the release of the proinflammatory cytokine tumor necrosis factor induced by strenuous exercise mediates adverse atrial remodeling and susceptibility to atrial fibrillation (65).

When considering various factors, it is crucial to make a comprehensive judgment. For athletes, despite the risks of high-intensity exercise causing arrhythmia, they tend to have a longer life expectancy than other AF patients due to a lower prevalence of metabolic diseases such as hyperlipidemia, diabetes, hypertension, and coronary heart disease (60). Meanwhile, the UK Biobank study also found that vigorous exercise in women reduces the risk of atrial fibrillation and fatal ventricular arrhythmias (52). This suggesting that the effects of exercise can vary among individuals, exercise intensity, frequency, and type should be adjusted according to a patient's unique metabolic and cardiovascular profile. According to the newest guideline of sports cardiology by ESC, although AF is more common among elite male athletes and those who engage in high-intensity endurance sports, it is reasonable to recommend moderate-intensity exercise to prevent AF because of the U-shaped curve between exercise and AF (66). Notably, metabolism-related assessments, such as thyroid function and drinking status, are essential prior to exercise. In patients combined with obesity, exercise intervention alone have little effect on fat mass, so the addition of resistance exercise to resistance exercise is recommended. This can improve the glucose tolerance, insulin sensitivity, lipid profile, and chronic inflammation (67–69). In patients combined with dyslipidemia, such as hypertriglyceridemia or hypercholesterolemia, more intense exercise is suitable because it can improve the lipid profile and reduce cardiovascular risk (70, 71). In patients combined with diabetes mellitus, both aerobic and resistance exercise enhance pancreatic function, with moderate- or high-intensity exercise having a more pronounced effect on reducing the risk of metabolic impairment. In addition, flexibility and balance exercises are beneficial, especially in patients with microvascular complications due to diabetes (72, 73).

High-Intensity Interval Training (HIIT) involves short, intense workouts that increase heart rate and burn more calories in a shorter period, creating a hypoxic state that boosts the body's oxygen demand. A study published in JAMA Network Open reports that twice-weekly, 23-minute HIIT sessions are as effective as twice-weekly, 60-minute moderate to high-intensity continuous training for improving functional capacity, resting heart rate, and quality of life in patients with atrial fibrillation (74). Here, HIIT is defined as two 8-minute interval training blocks of 30-second work periods at 80%-100% of peak power output interspersed with 30-second recovery. There can be many types of HIIT, as its no specific formula and there are no international guidelines, but an entire HIIT workout usually lasts between four and thirty minutes. HIIT is a cardiovascular exercise strategy that can have many types as its no specific formula and there are no universal international guidelines, but the entire HIIT workout usually lasts around thirty to forty minutes and is a combination of short repetitions of maximal-intensity exercise (anaerobic) followed by short bursts of lower-intensity activity (recovery) until fatigue, and both the number of repetitions and the length of each interval can vary even slight (75, 76). For example, there is also a typical 4 × 4 HIIT, which includes 4 min of exercise at 95% peak heart rate followed by 3 min of active recovery at 60%–75% peak heart rate, repeated four times (77, 78). HIIT patterns in existing studies vary due to the different fitness levels and tolerance of patients, which requires specific analysis by clinicians, rehabilitation therapists, and exercise and sport specialists (79). Importantly, this approach reduces the total amount of physical activity, lessening the patient's burden. Patients with atrial fibrillation often have dilated left atria and increased vagal tone, which decreases with age. Therefore, the effects of exercise vary based on age and should be recommended on a case-by-case basis (80). It is also crucial to acknowledge the differing impacts of exercise on men and women, requiring careful consideration by clinicians (81).

## 4 Effects of physical activity on cardiac metabolism

### 4.1 Physical activity and lipid metabolism

Considering the pathophysiologic mechanism of fat accumulation in the development of atrial fibrillation, regular exercise training, which is less intense than high-intensity training, may reduce the risk of cardiovascular incidents and is a safe and effective strategy (82). Physical activity improves physiological regulation in obese patients by enhancing blood flow and vascular function. Adopting a healthy lifestyle leads to reductions in plasma LDL, triglyceride, and cholesterol levels, which are significant contributors to cardiovascular disease susceptibility. During exercise, lipolysis and the level of free fatty acids increase. High fatty acid oxidation induces a protective phenotype that shields the myocardium from ischemia-reperfusion injury, providing cardioprotection during cardiac stress. However, psychosocial factors are also important. Obese individuals often exercise less due to psychological barriers and decreased respiratory muscle strength and ventilatory restrictions. Consequently, exercise not only reduces the risk of atrial fibrillation but also improves the quality of life for these patients. Metabolomics analyses have indicated that long-term exercise training significantly reduces lipid levels in animal models, suggesting a lipid response to exercise and pressure overload. Intensive lipidomic studies in cardiovascular disease have shown that lipid species in the heart, plasma, or serum can have potential prognostic value. In particular, changes in plasma phospholipids are strongly correlated with atrial disease (83).

### 4.2 Physical activity and glucose metabolism

Glucose oxidation can occur aerobically or anaerobically (glycolysis). Under conditions of relative hypoxia or certain pathological states, the body accelerates glycolysis. During exercise, the rate of aerobic glucose oxidation increases while the rate of glycolysis decreases. Aerobic glucose oxidation produces fewer protons, maintaining intracellular calcium ion balance, limiting calcium overload, preserving pH balance, and reducing energy expenditure. Thus, the metabolic phenotype generated during exercise is characterized by lower cardiotoxicity and enhanced cardioprotection (48). A study involving over 40,000 diabetes patients in South Korea demonstrated that individuals who had previously exercised but stopped and those who recently started exercising had a lower risk of developing atrial fibrillation compared to those who had not exercised for an extended period. The benefits of exercise for diabetics are potentially linked to improved vascular elasticity, offering protection against pathological atrial remodeling, oxidative stress, inflammation, and permanent damage (84, 85). However, the pressure and volume overload resulting from high metabolic demand during excessive exercise might adversely impact the atria (77, 86).

Although the exact pathophysiological mechanisms are not fully elucidated, structural and electrical remodeling are major contributors to AF. Potential mechanisms linking diabetes and atrial fibrillation include disturbances in energy metabolism, cellular calcium levels, ion channel function, and abnormal conduction, all of which depend on mitochondrial function.

### 4.3 Physical activity and secondary causes of atrial fibrillation

Secondary causes of AF can also impact on young adults or adolescent metabolism, such as hypertension, hyperthyroidism, lifestyle factors, alcohol consumption, smoking, cardiomyopathies and channelopathies. While exercise or physical activity has been shown to have many benefits, available data suggest an association between endurance exercise and atrial fibrillation and flutter (87), and some rare genetic diseases may also be triggered by exercise. For instance, AF is quite common in patients with hyperthyroidism (88), and men, aging, ischemic heart disease, congestive heart failure, and heart valve disease are associated with an increased risk of AF (89). Fortunately, study has shown a correlation between the amount of physical activity and changes in thyroid function in adults, including thyroid hormone levels and thyroid disorders (90). In addition, some rare arrhythmogenic diseases can cause AF and are closely related to physical exercise. For example, patients with long QT Syndrome, especially LQT3, are at increased risk for early AF (91). European guideline recommend that exercise should be avoided until the cause is effectively corrected (66). Similarly, athletes with catecholaminergic polymorphic ventricular tachycardia should undergo strict risk stratification, while maintaining a healthy lifestyle (92). Recent studies have shown that a generalized ban on exercise for all patients with hypertrophic cardiomyopathy is not justified, and that patients should undergo careful risk stratification and expert advice before choosing an implantable-cardioverter-defibrillator for treatment or exercise (66, 93). In conclusion, some secondary and genetic factors should also be taken into account when considering the effect of physical exercise on AF.

### 4.4 Physical activity and mitochondrial function

Maintaining normal mitochondrial function is crucial for muscle performance, and its deterioration is a hallmark of cardiovascular diseases. Long-term sustained aerobic exercise effectively preserves mitochondrial health, significantly contributing to long-term cardiovascular well-being. Enhancing the number and function of mitochondria in cardiomyocytes improves electron transfer and oxidative phosphorylation, reduces oxidative stress, promotes mitochondrial autophagy, increases muscle mass, and decreases apoptosis in cardiomyocytes. Regular exercise is a powerful countermeasure against mitochondrial depletion due to aging (94).

At the microscopic level, regulating mitochondrial fusion-associated proteins (Mfn2 and OPA1) and fission-associated proteins (DRP1) is a key target for cardiac therapy. Research indicates that inhibiting excessive mitochondrial fission benefits the heart, and exercise modulates changes in proteins related to mitochondrial fusion and fission (95, 96). Animal study has shown that aerobic training enhances impaired mitochondrial respiratory function and inhibits excessive fission while promoting fusion (97). During exercise, oxygen consumption and muscle ATP usage increase, leading to intensified ATP synthesis. Exercise training promotes endothelial nitric oxide synthase (eNOS)-dependent mitochondrial biogenesis in the heart, crucial for cardiac glucose transport. Coenzyme Q10, a component of the electron transport chain found in high-energy organs like the heart, increases in myocardial levels post-exercise. Elevated coenzyme Q10 levels are beneficial in conditions such as heart failure and diabetic cardiomyopathy (98). Thus, physical exercise positively impacts cardiac energy metabolism by influencing mitochondrial function (99).

### 4.5 Physical activity and the immune system

The relationship between physical activity and immunity, although not fully understood, exhibits significant heterogeneity based on the type, duration, and intensity of exercise. Physical activity rejuvenates the immune system and reduces inflammatory immune cells by regulating the circulation of immune progenitor cells (100, 101). While moderate exercise strengthens the immune system, strenuous exercise can be counterproductive (102). After vigorous exercise, a rise followed by a fall in peripheral blood lymphocytes is observed (103). The increase in lymphocytes is linked to the influx of natural killer cells and CD8+ T cells into the bloodstream, though the mechanism behind the post-exercise decrease in lymphocytes, potentially involving immune cell redistribution, remains unclear (104). Physical activity modulates macrophage function and regulates the expression of anti-inflammatory factors, possibly through the mTOR pathway or the NLRP3 inflammasome pathway (58). Beyond influencing cytokine synthesis and distribution, the type of muscle used in exercise and an individual's cardiorespiratory fitness level impact the entire immune system (105). Understanding how exercise influences immune cells both acutely and chronically is essential to complement current guidelines and optimize the therapeutic effects of exercise in preventing and reducing inflammation.

## 5 Discussion

With an increased focus on public health, preventive care, including physical activity and exercise, is gaining more attention. In cardiology, physical activity is linked to various beneficial effects, such as lowering blood pressure, improving hyperlipidemia, and increasing insulin sensitivity (106–109). Regular physical activity has been shown to reduce the risk of stroke and myocardial infarction, as well as decrease overall mortality risk (110). Additionally, regular exercise enhances atrial viability by increasing capillary density, preventing ischemia-reperfusion injury, and promoting the expression of antioxidant genes (111, 112). However, research findings can be contrasting. For example, a study on individuals with metabolic syndrome revealed that lifestyle changes, including diet and exercise, did not impact the occurrence of atrial fibrillation or alter the structure and function of the atria (113). Therefore, this review incorporates various studies to summarize and evaluate the actual effects of physical activity. Generally, the cardiovascular protective benefits of moderate exercise are well-established, partly due to improvements in the body's metabolic status.

A genome-wide association study involving 300,000 people indicated that a healthy lifestyle, including exercise, reduces the risk of developing cardiovascular disease, even in those with a genetic predisposition (114). This finding is significant, especially for individuals with high genetic susceptibility, as it underscores the potential for lifestyle improvements to confer substantial benefits.

This article first discusses the metabolic characteristics of patients with atrial fibrillation, including aspects such as glucose metabolism, lipid metabolism, energy metabolism, and inflammatory status. Understanding these specific metabolic characteristics is crucial for comprehending how exercise impacts these patients and exploring modifiable underlying mechanisms. Following this, the article reviews several clinical cohort studies to summarize the effects of different exercise patterns and intensities. It's important to recognize that more exercise is not always more beneficial, and individual differences must be considered.

During physical activity, the body's metabolic state undergoes changes in anabolism, catabolism, and energy metabolism. Physical activity helps maintain lower blood lipid and glucose levels, reducing the risk of cardiovascular disease, which is particularly crucial for individuals with atrial fibrillation. Exercise induces a protective phenotype through metabolic profile alterations and improves patients' lipid profiles. It also boosts energy metabolism, enhancing physical endurance and reducing frailty. Since glucose and fatty acids are substrates for ATP production, there is a mutual influence between energy metabolism and material metabolism.

Additionally, physical activity positively influences the heart's structure and function in patients with atrial fibrillation. Moderate exercise can enhance heart rhythm and decrease the frequency of atrial fibrillation episodes. Establishing proper exercise habits can improve heart structure and function, reducing the risk of cardiac hypertrophy and, consequently, the incidence of atrial fibrillation. Furthermore, the social and psychological aspects are equally important. Exercise not only benefits physical health but also alleviates psychological issues like anxiety and depression, enhancing the quality of life. Engaging in sports activities increases social interaction and bolsters patients' social support, fostering a supportive community environment for patients.

This review also offers suggestions for future research and clinical practice. There is a growing consensus among clinicians and researchers to employ physical activity as a preventive and therapeutic approach for various heart diseases, including heart failure and atrial fibrillation. These interventions are straightforward, typically without notable side effects, and can reduce healthcare costs while enhancing overall population health. Importantly, a tailored exercise prescription should be provided, considering the individual's condition and characteristics, aligning with the direction of precision medicine.

In conclusion, physical activity benefits not only patients with atrial fibrillation but also those with other cardiovascular diseases, largely through metabolic improvement. Efforts are needed to alter patients' misconceptions and physical limitations regarding exercise, promoting its benefits while ensuring safety and personalization. However, this review acknowledges its limitations, as sports cardiology is an evolving field. The relevant foundational research is not yet extensive, and more detailed findings are required to substantiate the arguments presented.
